# Effect of Drying Conditions and Jojoba Oil Incorporation on the Selected Physical Properties of Hydrogel Whey Protein-Based Edible Films

**DOI:** 10.3390/gels10050340

**Published:** 2024-05-17

**Authors:** Sabina Galus, Magdalena Karwacka, Agnieszka Ciurzyńska, Monika Janowicz

**Affiliations:** Department of Food Engineering and Process Management, Institute of Food Sciences, Warsaw University of Life Sciences, Nowoursynowska Str. 159c, 02-776 Warsaw, Poland; agnieszka_ciurzynska@sggw.edu.pl (A.C.); monika_janowicz@sggw.edu.pl (M.J.)

**Keywords:** hydrogels, edible films, whey protein isolate, jojoba oil, drying

## Abstract

Edible hydrogel coatings or films in comparison to conventional food packaging materials are characterized as thin layers obtained from biopolymers that can be applied or enveloped onto the surface of food products. The use of lipid-containing hydrogel packaging materials, primarily as edible protective coatings for food applications, is recognized for their excellent barrier capacity against water vapor during storage. With the high brittleness of waxes and the oxidation of different fats or oils, highly stable agents are desirable. Jojoba oil obtained from the jojoba shrub is an ester of long-chain fatty acids and monovalent, long-chain alcohols, which contains natural oxidants α, β, and δ tocopherols; therefore, it is resistant to oxidation and shows high thermal stability. The production of hydrogel films and coatings involves solvent evaporation, which may occur in ambient or controlled drying conditions. The study aimed to determine the effect of drying conditions (temperature from 20 to 70 °C and relative humidity from 30 to 70%) and jojoba oil addition at the concentrations of 0, 0.5, 1.0, 1.5, and 2.0% on the selected physical properties of hydrogel edible films based on whey protein isolate. Homogenization resulted in stable, film-forming emulsions with bimodal lipid droplet distribution and a particle size close to 3 and 45 µm. When higher drying temperatures were used, the drying time was much shorter (minimum 2 h for temperature of 70 °C and relative humidity of 30%) and a more compact structure, lower water content (12.00–13.68%), and better mechanical resistance (3.48–3.93 MPa) of hydrogel whey protein films were observed. The optimal conditions for drying hydrogel whey protein films are a temperature of 50 °C and an air humidity of 30% over 3 h. Increasing the content of jojoba oil caused noticeable color changes (total color difference increased from 2.00 to 2.43 at 20 °C and from 2.58 to 3.04 at 70 °C), improved mechanical elasticity (the highest at 60 °C from 48.4 to 101.1%), and reduced water vapor permeability (the highest at 70 °C from 9.00·10^−10^ to 6.35·10^−10^ g/m·s·Pa) of the analyzed films. The observations of scanning electron micrographs showed the heterogeneity of the film surface and irregular distribution of lipid droplets in the film matrix.

## 1. Introduction

There is a great interest in finding new opportunities to reduce packaging waste, which constitutes a large share of the total waste [1,2]. Most food products are sold in unit packaging, much of which is made from synthetic materials. An alternative to this packaging is biodegradable polymers [2,3,4]. Films made from natural polymers (proteins, polysaccharides, and fats) are promising options for partially replacing plastic unit packaging [5,6,7]. Coatings are produced directly on the product surface by immersion and then dried. Films, on the other hand, are produced separately from the product and may serve as a barrier separating individual product components or act as independent packaging when used [8,9]. Currently, disposable, soluble sachets for loose and dry products (sugar, instant coffee, cocoa, herbs, and spices) can be made from protein or polysaccharide films. Janjarasskul et al. [10] developed completely water-soluble, odorless, and visually clear edible pouches from whey protein isolate that showed good instant coffee protection. Similarly, Hromiš et al. [11] analyzed composite pumpkin oil cake and duplex pumpkin oil cake/maize zein packaging films as a potential protective pouches for packing flaxseed oil. The authors noted that developed films ensure good oxidative stability and mitigate the less satisfactory sensory quality of oil. In addition, significance changes in oil composition were not observed.

Whey proteins are a fraction of milk protein that includes β-lactoglobulin and α-lactalbumin. They are produced in large quantities and recovered from whey on a large scale through ultrafiltration, ion exchange, and electrophoresis [12,13]. These proteins are characterized by excellent nutritional and functional properties, such as solubility and the ability to thicken, gel, and create emulsions and foams, making them suitable for producing edible coatings and films [14,15]. Hydrogel films based on milk proteins are characterized by good barrier properties to oxygen, flavors, and lipids as well as good mechanical properties [10,16]. However, due to the hydrophilic nature of whey proteins, coatings made from them exhibit a low barrier to water vapor [17]. Their composition also contributes to the enhancement of the nutritional properties of the coated food. Whey proteins have been used in different applications, including fruits such as apples [18] or plums [19] and vegetables such as broccoli [20] as well as in prolonging the shelf life of cheeses [12,21].

The use of protective edible coatings containing lipids as well as different functional substances has been known for centuries, particularly in preserving citrus fruits from water loss [22]. Due to the high brittleness of wax coatings, new forms of coating mixtures have been sought [23]. Dispersing a fatty substance in water or another solvent creates an emulsion that performs a protective function when applied to the product [24]. However, lipids are prone to oxidation, which significantly reduces the durability of the coating layers [25]. Therefore, a lipid substance with a low degree of oxidation is desirable as a component of coating mixtures. Jojoba oil, obtained from the jojoba shrub, is an ester of long-chain fatty acids and long-chain alcohols, containing natural antioxidants α, β, and δ tocopherols, making it resistant to oxidation. It is a wax that remains fluid at room temperature and has high thermal stability (up to 300 °C), giving it great potential for practical applications [26,27,28]. In addition, jojoba oil exhibited an antibacterial effect against selected microbes such as *Escherichia coli*, *Klebsiella species*, and *Staphylococcus aureus*. Therefore, this oil can be used as a natural source of preservatives in food to combat the selected bacterial species responsible for food-borne diseases or food spoilage [29,30]. The film-forming technique involves the evaporation of the solvent (usually water), which can occur under ambient conditions or during controlled drying, significantly shortening this time [31,32,33,34]. The production of biopolymer packaging films or hydrogel edible coatings directly on products in industrial settings requires a brief drying period. The temperature and relative humidity of the environment are crucial and affect the functional properties of coating layers, and determining this influence is important in designing film or coating technology [35,36,37]. Moreover, the stability of edible films depends on their mechanical properties and moisture barrier characteristics. These properties are strongly influenced by the presence of water, which is affected by drying and storage conditions [38].

The study aimed to prepare hydrogel packaging films based on whey protein isolate incorporated with jojoba oil at the concentrations 0, 0.5, 1.0, 1.5, and 2.0% and evaluate the effect of drying conditions, such as temperature from 20 to 70 °C and relative humidity from 30 to 70%, and oil concentration on the water content, color (L*, a*, b*, and ΔE), mechanical properties (tensile strength and elongation at break), water vapor permeability, and microstructure of the films. The particle size and distribution of hydrogel emulsions were also characterized. 

## 2. Results and Discussion

### 2.1. Whey Protein-Based Hydrogel Film Characterization

Oil-in-water emulsions based on whey protein isolate and jojoba oil at four concentrations—0.5%, 1%, 1.5%, and 2%—were prepared using homogenization. The solutions were then poured and dried to obtain hydrogel films. Emulsification is a crucial technique for obtaining a mixture of two substances with different characteristics, such as water and oil. A low concentration of oil particles has a relatively positive effect when the dispersion is steady and, in most cases, stabilized by a biopolymer [22]. The control samples were films without the addition of jojoba oil. All the obtained hydrogel whey protein films with jojoba oil were visually homogeneous, without cracks or pores. There was no macroscopic change in films, as a smooth surface and flexible structure were observed. After the drying process, a certain amount of oil was observed on the surface of the films. A homogeneous surface on the support side (Petri dishes) and a slightly rough surface on the air side were observed for each film. With increasing concentrations of oil, an increased amount of lipid was observed on the surface of the films. Probably, more lipid droplets were concentrated on the air side, which drove the particle toward the film surface with the migration of the solvent during the drying-induced changes. Similar observations were noted for biopolymer films with the addition of different vegetable oils in our previous works [39]. The analyzed whey protein films were opaque and matte in contrast to the control films, which were transparent and slightly shiny. Shaw et al. [40] observed dullness and opacity in their studies on the influence of soybean oil emulsion on the mechanical properties of whey protein-based films.

### 2.2. Jojoba Oil Droplet Particle Size and Distribution in Hydrogel Whey Protein Film-Forming Solutions

**Figure 1:**
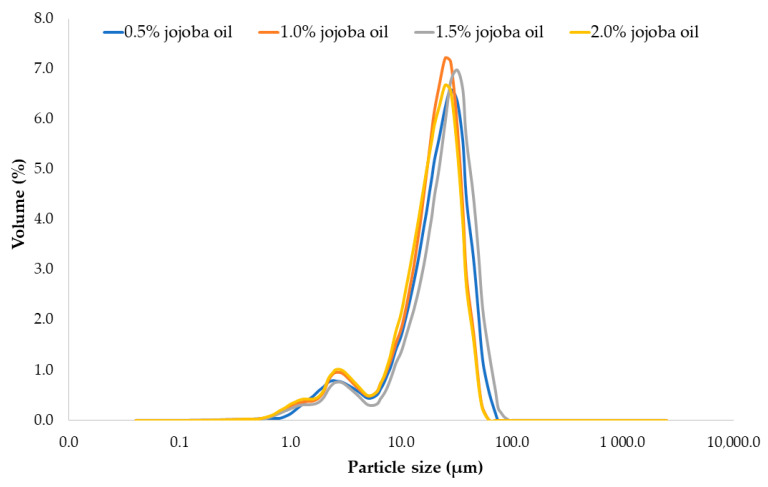
Particle size distribution in soy protein film-forming solutions with jojoba oil (JO).

Homogenization is a process that simultaneously fragments and homogenizes particles of the dispersed phase within the continuous phase. This technique aims to increase the stability of the structure of the processed system [41,42]. Factors influencing the effectiveness and advisability of homogenization include pressure, homogenization temperature, oil phase concentration, and length of the homogenizing gap [43]. In general, an increase in pressure positively affects the homogenization effect, expressed as the average diameter of lipid droplets. Temperature affects the degree of lipid dispersion and their tendency to form aggregates after homogenization as well as determines the color of the emulsions. In addition, an increase in the concentration of the lipid phase reduces the homogenization effect. Therefore, the size of emulsion droplets is fundamentally important because it affects the rheological properties of the emulsion and its derivative products. Quantitative measures such as the degree of dispersion and the number of fractions are also employed to characterize the properties of multiphase systems. Presently, the laser diffraction method is utilized to measure these quantities, and this technique is commonly applied to analyze particle sizes ranging from 0.5 to 1000 microns. In general, greater homogeneity of film-forming emulsions and smaller lipid globule sizes reduce water vapor permeability [22]. The results of particle size and distribution of film-forming solutions with 0.5–2% jojoba are presented in Figure 1. 

The particle size analysis indicates that the size distribution of oil droplets across all jojoba oil concentrations (0.5–2%) was remarkably consistent. An increasing concentration of jojoba oil in the film-forming solutions did not compromise the stability of the tested multiphase emulsion. In the tested samples, no significant differences were found in the size distribution of oil droplets (*p* < 0.05). Their size for all four fractions ranged from 3.10 to 45.21 µm, with the average size ranging from 18.86 to 25.49 µm, and their state of aggregation remained consistent. According to Domian [44], these observations confirm the stability of the obtained emulsion. Due to the surface properties, milk proteins play a primary function as emulsion-stabilizing colloids. The phenomenon of emulsion stabilization by proteins is determined by their ability to reduce surface tension, adsorb at the phase boundary, and create a coherent layer around oil droplets. Hogan et al. [45] observed spray-dried emulsions with a dispersed phase of soybean oil ranging from 0.25 to 3.0 relative to the protein component (corresponding to the oil content in powdered emulsions from 20 to 75%), and it was concluded that the size of the oil droplets was independent of the protein-to-oil ratio. The authors also noted that the reason for the lack of dependence of oil droplet size on the oil-to-protein ratio in the tested system was due to the higher total protein concentration exceeding its critical micellar concentration.

### 2.3. Effect of the Temperature and Relative Humidity on the Drying Time of Hydrogel Whey Protein Film-Forming Solutions

The drying process directly affects the quality of produced coatings and films [36]. The most significant physical changes occurring during the drying of edible coatings and films are volume reduction and structure stiffening due to water evaporation [37]. The drying method and conditions such as temperature, air humidity, and drying time play crucial roles in this process. They influence not only the physicochemical changes and quality of the hydrogel materials but also the costs of the entire process. The optimal selection of drying parameters is extremely important for processes performed on an industrial scale. Other equally important factors encouraging manufacturers to develop the most optimal conditions for the drying process include external requirements related to stricter safety and environmental protection standards as well as the development and availability of new technologies. The use of appropriate drying conditions and methods reduces the energy consumption of the process, improves its implementation, and lessens the impact on the environment. When selecting drying conditions and a device, it is important to ensure that the obtained products are neither too dry nor too moist. To determine the most optimal drying conditions, the obtained film-forming solutions were subjected to a drying process at temperatures of 20, 30, 40, 50, 60, and 70 °C; a constant relative humidity of 50%; a constant temperature of 50 °C; and a relative humidity of 30, 40, 50, 60, and 70% in the climatic chamber. The drying time was measured for each parameter, and the results are summarized in Table 1. Research indicated that temperature and relative humidity significantly affect the drying time of whey protein films (*p* < 0.05). Observations showed that the drying time of film-forming solutions decreased from 18 h (temperature of 50 °C and relative humidity of 70%) to 2 h (temperature 70 °C and relative humidity of 30%) with an increase in temperature. At higher temperatures (40, 50, 60, and 70 °C), drying times ranged from 2 to 4 h. The most significant changes in drying time occurred at temperatures of 20, 30, and 40 °C (*p* < 0.05). Kurek et al. [35] observed that drying chitosan films produced in acetic acid solution (1–3%) at temperatures of 20, 60, and 100 °C with a relative humidity of 30% exhibited certain relationships. The authors noted that the drying time decreased as the temperature increased, with the longest drying time occurring at 20 °C (14 to 18 h depending on the concentration of acetic acid) and the shortest times at 60 and 100 °C (0.75 to 4.5 h). Similar observations were presented for pullulan, alginate, and blended films dried at 40, 50, or 60 °C [46]. These reports suggest that changes in drying temperature significantly impact the rate of solvent evaporation as well as solute immigration. Moreover, this can ultimately influence the physicochemical properties of the packaging films. Therefore, changes in the duration of the drying process significantly control the physical and chemical attributes of the films [36].

A shrinkage of the whey protein film structure and corrugation of their surface was observed at a temperature of 70 °C as a result of different changes that can be attributed to the nature of milk proteins. It suggests that the duration of the drying process also depends on the type of biopolymers used for film preparation. The effect of relative humidity in the drying chamber influenced the drying time. The increase in air humidity from 30% to 70% at a temperature of 50 °C contributed to an increase in the drying time of the films from 3 h to 18 h. The films took the longest to dry at 60% and 70% air humidity (15 and 18 h, respectively), and the shortest at 30% humidity (3 h). The thickness of the obtained films was around 130 ± 10 µm, with a tendency toward higher values as the amount of oil increased. However, these differences were not statistically significant (*p* < 0.05). Therefore, jojoba oil did not affect the drying time of hydrogel whey protein films. Analyzing the influence of temperature and relative humidity on drying time revealed that the optimal conditions for drying films based on whey protein isolate are a temperature of 50 °C and an air humidity of 30% over 3 h.

### 2.4. Effect of Drying Conditions and Jojoba Oil on the Water Content of Hydrogel Whey Protein Films

**Figure 2:**
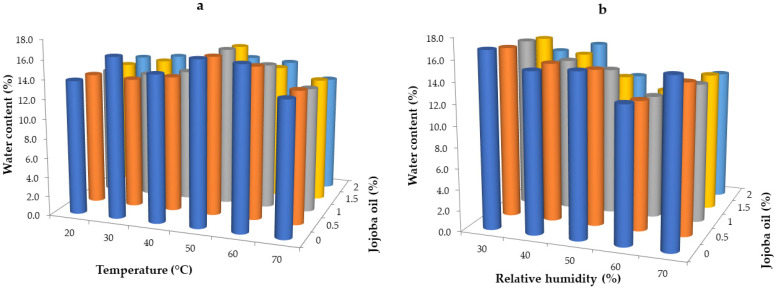
Water content in whey protein films with the addition of jojoba oil dried in variable temperature conditions at a relative humidity of 30% (**a**) and variable relative humidity conditions at the temperature of 50 °C (**b**).

Water acts as a plasticizer and its content greatly influences the properties of these coatings and films [47]. In general, an increase in water content results in a significant increase in water activity, while a decrease in water content significantly reduces water activity. Water content in food products determines the degree to which water molecules are associated with food ingredients. It also affects physical and chemical changes, which impact the quality and shelf life of products. Regarding hydrogel films and coatings, water molecules can be easily lost from coatings during drying and storage in environments with low relative humidity, thereby causing deterioration of their elasticity [38]. The stability of edible films is a component of their mechanical properties and moisture barrier properties. These properties are strongly dependent on the presence of water, the content of which is influenced by drying and storage conditions [46]. To determine the effect of drying conditions such as temperature and relative humidity combined with jojoba oil concentration on the water content of the hydrogel whey protein films, samples with 8% whey protein isolate were dried at 105 °C for 24 h. The percentage of water content was calculated based on the weight differences of the samples before and after drying. The results are presented in Figure 2.

An increase in the drying temperature from 20 to 70 °C did not significantly affect the water content in whey films (*p* < 0.05) (Figure 2a), although films dried at 50 and 60 °C had a higher water content than those dried at 20, 30, and 40 °C. The lowest values of water content were noted for film dried at 70 °C, which is probably attributed to short drying time and fast water evaporation, resulting in shrinkage. Films dried at 50 °C had the highest water content, ranging from 13.80 to 16.81%, depending on the addition of jojoba oil. Very similar values were also obtained for films dried at 60 °C, ranging from 13.55 to 16.64%. Based on the results obtained, it was found that whey films dried at a temperature of 70 °C and a relative humidity of 30% were characterized by the lowest water content percentage, ranging from 12.00 to 13.68%. Films dried at 20 °C had a similar water content (from 12.99% to 13.77%). Despite obtaining films with the lowest water content at 70 °C, this is not the most favorable drying temperature due to shrinkage and lower functional properties of whey films. Similar conclusions were reported by Jakubczyk and Pokrzywnicki [48], who examined the effect of drying temperature on the water content in a model agar gel. Apart from drying temperatures of 50 and 80 °C, the authors did not observe any significant differences in water content. Soazo et al. [49] studied the effect of drying temperature and the addition of beeswax on whey films. They found that materials dried at 5 °C had lower equilibrium moisture content than those dried at 25 °C. This was observed in films with both 0% and 40% beeswax content; however, for films with 20% beeswax, the effect was the opposite. According to Jakubczyk and Pokrzywnicki [48], drying temperatures above 60 °C influence the hardening of materials, which in turn affects the deterioration of their mechanical properties. The relationships described were largely determined by drying time. The results indicate that the drying time had an impact on the water content in whey films. Films dried for 17 h at 20 °C and 30% relative humidity showed a similar water content to those dried for 2 h at 70 °C and 30% relative humidity. The lack of significant differences in water content, despite the increase in drying temperature, was due to decreasing the drying time from 17 to 2 h.

The effect of relative humidity and jojoba oil content on the water content of whey films is presented in Figure 2b. An increase in relative humidity during drying from 30% to 60% typically results in a decrease in water content within the films. However, at a relative humidity of 70%, this downward trend is different. Whey films dried at a temperature of 50 °C and a relative humidity of 70% exhibited an increased water content compared to those dried at relative humidities of 50% and 60%. The lowest water content was found in films dried at a relative humidity of 60% (ranging from 11.54 to 12.94%, depending on the addition of jojoba oil). Films dried at 50 °C and 30% relative humidity had the highest water content (ranging from 13.80 to 16.81%). The described relationships, similar to the effect of temperature on water content, were also largely determined by the drying time. The results indicate that the drying time impacted the water content in the whey films. The decrease in the percentage of water content, despite the increase in relative humidity of the environment, was due to the increase in drying time (from 3 to 18 h). Based on the analysis of the results presented in Figure 2a,b, it was found that an increase in the concentration of jojoba oil (from 0 to 2.0%) resulted in a slight reduction in the water content of the tested whey films. This relationship was noted when examining both the effect of relative humidity and the drying temperature on the water content of the obtained films. Moreover, control samples without jojoba oil addition had the highest water content across all drying parameters. This phenomenon is attributed to the character of jojoba oil as a hydrophobic substance in the hydrocolloid matrix of the films, leading to a modification in the water content within the film structure [14,50,51]. Soazo et al. [49] noted similar conclusions in their research on whey films, finding that adding 20% and 40% beeswax significantly reduced the equilibrium moisture content of the films tested. Kim and Ustunol [52] also reported that lipid addition to whey protein isolate films plasticized with sorbitol and glycerol reduces equilibrium humidity. However, Zinoviadou et al. [53] observed that low concentrations of oregano oil slightly reduced the water content in whey protein isolate-based films. Nevertheless, interactions between lipids and the biopolymer matrix combined with the drying conditions affect the physicochemical characterization of hydrogel films.

### 2.5. Effect of Drying Conditions and Jojoba Oil on the Color of Hydrogel Whey Protein Films

**Figure 3:**
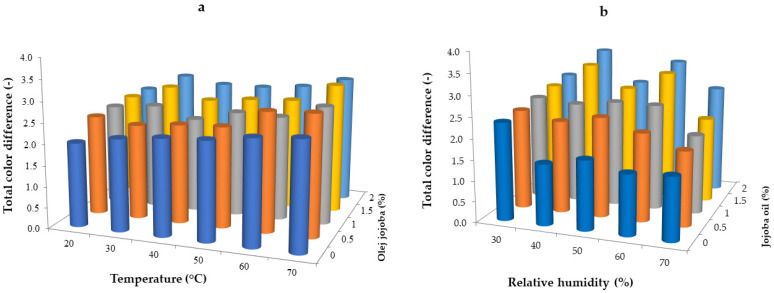
Total color difference of whey protein films with the addition of jojoba oil dried in variable temperature conditions at a relative humidity of 30% (**a**) and variable relative humidity conditions at a temperature of 50 °C (**b**).

Edible films and coatings must be as colorless as possible for food application; however, when applied as pH-sensitive intelligent indicators, suitable color is desired [54,55,56]. In general, color is important when assessing the freshness, suitability, and attractiveness of a given product. It is one of the most important indicators that determine the consumer’s choice of product. Color measurement is also used when assessing the quality of many technological processes, including drying, osmotic dehydration, and expansion processes [57]. To determine the color changes of whey coatings influenced by varying drying conditions (temperature and relative humidity) and the addition of jojoba oil, the films were measured using a colorimeter in the CIE L*a*b* color system. Here, L* represents lightness, while a* and b* are the chromaticity coordinates. To interpret the results, a total color difference (ΔE) is used to describe how much the tested material’s color deviates from the standard. Figure 3 shows the results of the color difference (ΔE) of the analyzed hydrogel whey protein films. The changes in drying temperature from 20 to 70 °C did significantly affect the ΔE parameter values (*p* < 0.05) (Figure 3a). An increasing trend in results was observed with increasing drying temperatures. The drying parameters with the lowest ΔE values were a temperature of 20 °C and relative environmental humidity of 30% (from 2.00 to 2.43). The highest values of the ΔE parameter were observed at a temperature of 70 °C and a relative environmental humidity of 30% (2.58 to 3.04). Analyzing the effect of jojoba oil on changes in the ΔE parameter, it was found that its addition caused significant changes (*p* < 0.05), with a tendency for the parameter to increase under the influence of increasing concentrations of jojoba oil. Galus and Lenart [58] observed a significant color change by studying the impact of lipid emulsion addition on the optical properties of whey protein films. An increase in the total color difference value as the result of the addition of rapeseed oil, pumpkin seed oil, and hazelnut oil to whey films was also noted [59]. The drying process and the addition of jojoba oil significantly affected the color of the hydrogel whey protein films. These changes are noticeable even to an inexperienced observer, according to the International Commission on Illumination. This criterion shows that total color difference values in the range of 0–2 are not recognizable by humans; those in the range of 2–3.5 are recognizable by an inexperienced observer; and those >3.5 show noticeable, clear differences in color deviation [60]. The drying process resulted in whey films with variable color characteristics. During the visual assessment, it was observed that the control films without the addition of jojoba oil were shinier and more transparent than those with jojoba oil, which were characterized by matte texture and a slightly darker, yellowish color. Similar observations were obtained for whey films with the addition of soybean oil [61] and rapeseed oil [50].

Different relative humidities of the environment during drying did not significantly affect the total color difference (ΔE) (*p* < 0.05). The drying parameters with the lowest ΔE values corresponded to a relative humidity of 70% at a temperature of 50 °C, ranging from 1.51 to 2.61. The highest values of the ΔE parameter were observed at a relative environmental humidity of 30% and a temperature of 50 °C, ranging from 2.35 to 2.7. Analyzing the effect of jojoba oil on the ΔE parameter under variable environmental relative humidity conditions, it was found that the addition of jojoba oil caused significant changes in the ΔE parameter (*p* < 0.05). There was a noticeable tendency for the parameter to increase under the influence of increasing concentrations of jojoba oil. Undoubtedly, films dried in different humidity conditions exhibited lower values of the ΔE parameter than those dried in different temperature conditions. The conclusion is that drying temperature has a much greater impact on the total color difference than the relative humidity. In both cases, i.e., temperature or relative humidity ranges, the lowest values of total color differences were observed in control samples without the addition of jojoba oil.

### 2.6. Effect of Drying Conditions and Jojoba Oil on the Mechanical Properties of Hydrogel Whey Protein Films

**Figure 4:**
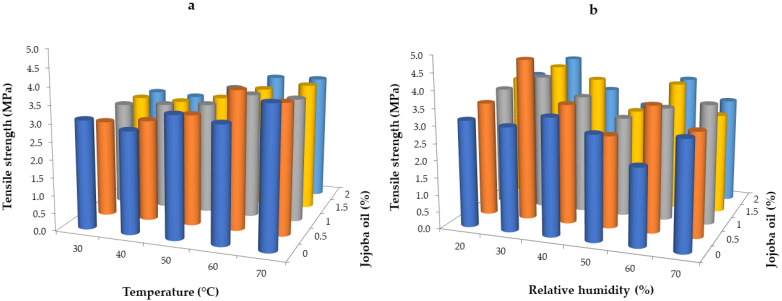
Tensile strength of whey protein films with the addition of jojoba oil dried in variable temperature conditions at a relative humidity of 30% (**a**) and variable relative humidity conditions at the temperature of 50 °C (**b**).

Mechanical properties are crucial for the application of edible films and coatings on food products because they mitigate various defects affecting the appearance, shelf life, and mechanical properties of food items. Mechanical property analysis typically involves measuring two parameters such as tensile strength, which is the maximum force required to tear the film, and elongation at break, which is the maximum distance to which the film has been stretched. The results of the tensile strength for hydrogel whey protein films are presented in Figure 4. Films dried at temperatures from 20 to 70 °C in a constant relative humidity of 30% showed the tendency to higher values at higher temperatures (Figure 4a). The values were in the range of 2.71 to 3.93 MPa. The lowest values were observed for films dried at 30 °C and the highest for those dried at 70 °C. This is probably due to the film format during drying and the mechanism of water evaporation, which was much faster at higher temperatures. In addition, a shrinkage was observed for the highest temperature; thus, large changes in the structure occurred, influencing the tensile strength. Popovic et al. [62] dried films under variable temperature conditions (70–90 °C) and variable pH conditions (10–12). After measuring the tensile strength of the obtained films, they found that an increase in temperature at the same pH increased tensile strength (from 15.08 to 47.19 MPa at pH 11).

The addition of jojoba oil resulted in lower values, indicating that the oil droplets softened the film structure and showed lower mechanical resistance. According to Yang and Paulson [63], introducing lipids into coatings means enhancing their barrier properties; however, these ingredients negatively affect optical and mechanical properties. Similar observations were noted by others for biopolymer-based packaging films [64,65,66].

The results presented in Figure 4b indicate that the relative humidity did not significantly affect the tensile strength of the whey films (*p* < 0.05). The results were in the range of 2.72–4.70 MPa. Films dried at a relative humidity of 30% exhibited the highest strength (3.04–4.70 MPa), while those dried at 50% had the lowest(2.71–3.06 MPa), despite achieving the highest tensile strength values. The results for other relative humidities remained relatively constant with the tendency of higher values at higher relative humidity. This is due to the plasticizing effect of water molecules in humid conditions, resulting in a more soft structure and water remaining in the film matrix [38].

**Figure 5:**
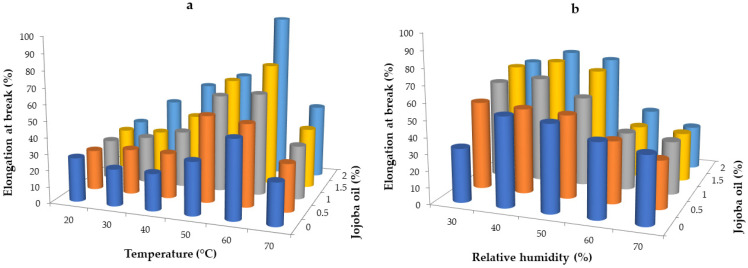
Elongation at break of whey protein films with the addition of jojoba oil dried in variable temperature conditions at a relative humidity of 30% (**a**) and variable relative humidity conditions at the temperature of 50 °C (**b**).

The effect of the drying temperature and the relative humidity on the elongation at break is presented in Figure 5. The results were between 22.60 and 101.10%. The research shows that temperatures of 50 °C and 60 °C significantly influenced the elongation at break of films obtained under these drying conditions (*p* < 0.05) (Figure 5a). The values were in the range 32.60–61.70% and 48.40–101.10%, respectively. The remaining drying temperatures (20, 30, 40, and 70 °C) were characterized by similar values of elongation at break, ranging from 22.60 to 45.00%. Notably, temperatures between 20–60 °C had a significant impact on increasing the elongation of coatings with a 2% jojoba oil content (from 24.3 to 101.1%). The addition of jojoba oil extended the whey films, and an increase in film elongation at break was observed with higher jojoba oil concentrations. These changes were particularly noticeable at drying temperatures of 40, 50, and 60 °C.

The research shows that the relative humidity significantly affected the elongation at break of hydrogel whey protein films (*p* < 0.05) (Figure 5b). As the relative humidity decreased (from 70 to 30%), the elongation values of the films increased. The highest elongation values were recorded for films dried at a relative humidity of 30 and 40% (ranging from 32.59 to 70.00%), while the lowest values were observed for films obtained at a relative humidity of 60 and 70% (ranging from 31.67 to 45.00%). The addition of jojoba oil extended the whey films’ elongation. For films obtained at a relative humidity of 30–50%, an increase in elongation was observed with increasing concentrations of jojoba oil, with the changes being particularly noticeable at concentrations of 1.0–2.0%. For films obtained at relative humidities of 60% and 70%, a tendency for the elongation value of the coatings to decrease was observed with increasing jojoba oil concentration. This is due to the plasticizing effect of jojoba oil and the less compact film structure.

Numerous studies have examined the impact of various factors on the mechanical properties of edible films. Shaw et al. [61] noted that the tensile strength of whey protein films depends on their lipid content. The authors showed an initial reduction in tensile strength at low concentrations of soybean oil, followed by a slight increase at higher concentrations. Monedero et al. [67] showed a significant reduction in tensile strength in soy coatings with the addition of a fatty mixture of oleic acid and beeswax. Heating affects the mechanical properties by significantly increasing tensile strength while reducing elongation. Similarly, ultraviolet radiation alters the mechanical properties of films in a manner comparable to increased temperature. Moreover, both tensile strength and elongation at break strongly depend on the plasticizer type and concentration [40]. Glycerol, which was used in this study for the film preparation, is a hydrophilic compound [38] that provides a similar character to biopolymer films plasticized with glycerol.

### 2.7. Effect of Drying Conditions and Jojoba Oil on the Water Vapor Permeability of Hydrogel Whey Protein Films

The water vapor barrier properties of hydrophilic films or coatings, including whey protein films, depend mainly on the solubility and diffusivity of water molecules in the substance constituting the film matrix. Both materials containing lipids are characterized by lower water vapor permeability due to their hydrophobic nature. In contrast, films and coatings based on hydrophilic polymers are very sensitive to water and exhibit excessive water vapor permeability [22]. Water vapor barrier is a feature that largely determines the functionality of the coating because changes in the humidity of a food product determine its freshness [68]. In general, the ability of water vapor to easily penetrate structures based on proteins and the presence of glycerol in these structures does not constitute an effective barrier. Water vapor permeability through biopolymeric films is influenced by many factors, such as the nature of the biopolymer and plasticizer, drying conditions, the addition of other functional compounds. Films with glycerol have much higher water vapor permeability than those plasticized with sorbitol [40]. In addition, the heating temperature film-forming solutions influence their physicochemical properties, including water vapor permeability.

**Figure 6:**
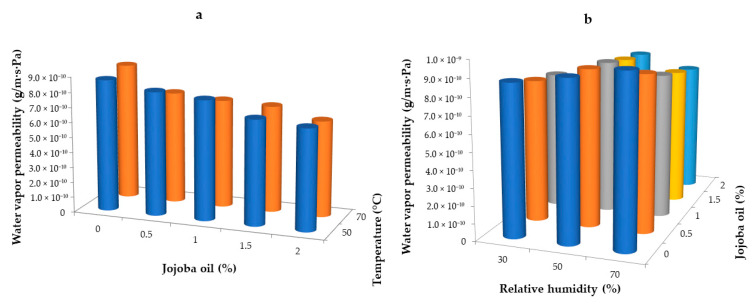
Water vapor permeability of whey protein films with the addition of jojoba oil dried in variable temperature conditions at a relative humidity of 30% (**a**) and variable relative humidity conditions at a temperature of 50 °C (**b**).

To determine whether water vapor permeability is affected by the temperature and relative humidity of the environment during drying and the addition of jojoba oil, selected samples of whey films were tested, and the results are presented in Figure 6. The tests involved films dried at temperatures of 50 °C and 70 °C with a relative humidity of 30% (Figure 6a) and those dried at 50 °C with relative humidity levels of 30%, 50%, and 70% (Figure 6b). Both figures show the relationship between water vapor permeability, drying temperature, and the addition of jojoba oil. The statistical analysis confirmed that the drying temperature did not significantly affect the water vapor permeability values (*p* < 0.05). However, a slight increase in these values was observed for coatings dried at 50 °C with a relative humidity of 30%, which may be attributed to a higher water content in the films. An increase in the concentration of jojoba oil results in a slight decrease in water vapor permeability, significantly influenced by its hydrophobic nature. The slight differences may have been caused by minimal variations in jojoba oil concentrations; however, they were noticeable. Control samples without the addition of jojoba oil exhibited the highest water vapor permeability, with values of 8.71·10^−10^ g/m·s·Pa at 50 °C and 9.00·10^−10^ g/m·s·Pa at 70 °C, respectively. The addition of 2.0% jojoba oil reduced the water vapor permeability values to 6.70·10^−10^ g/m·s·Pa for 50 °C and 6.35·10^−10^ g/m·s·Pa for 70 °C. The introduction of lipids into hydrophilic biopolymer-based films, including whey protein films, allows modification of their barrier and protective properties. The physical state of lipids significantly impacts the barrier properties of films, as water has a greater affinity for liquid lipids than solid ones [5,14].

The analysis of the data showed the dependence of water vapor permeability on the relative humidity of the environment and the addition of jojoba oil, indicating that the relative humidity influences changes in water vapor permeability values, although no clear trend is observed (Figure 6b). The obtained data showed that the lowest water vapor permeability was observed for films dried at a temperature of 50 °C and relative humidity of 30%, ranging from 6.7·10^−10^ g/m·s·Pa to 8.71·10^−10^ g/m·s·Pa. Films dried at a temperature of 50 °C with a relative humidity of 50% showed a tendency to obtain higher values from 8.31·10^−10^ g/m·s·Pa to 9.19·10^−10^ g/m·s·Pa and from 7.56·10^−10^ g/m·s·Pa to 9.77·10^−10^ g/m·s·Pa, respectively. Research conducted by Kurek et al. [35] indicated that an increase in the relative humidity of the environment results in the deterioration of the barrier properties of chitosan films, with or without the addition of carvacrol. For higher values of relative environmental humidity (30–75% and 30–100%), the authors observed slight differences in water vapor permeability. An increase in the concentration of jojoba oil under variable environmental relative humidity conditions (30–70%) slightly reduced the water vapor permeability value, significantly influenced by its hydrophobic nature. The slight differences could have been due to small differences in jojoba oil concentrations, yet they were noticeable. Control samples, without the addition of jojoba oil, for the three drying parameters tested were characterized by the highest water permeability. The results were 8.71·10^−10^ g/m·s·Pa, 9.19·10^−10^ g/m·s·Pa, and 9.77·10^−10^ g/m·s·Pa for 30, 50, and 70% relative humidity, respectively. The addition of jojoba oil at 2.0% reduces the water vapor permeability values to 6.7·10^−10^ g/m·s·Pa for 30% relative humidity, to 8.31·10^−10^ g/m·s·Pa for 50%, and to 7.56·10^−10^ g/m·s·Pa for 70%. Furthermore, lower water vapor permeability values were observed under variable temperature conditions than under variable environmental relative humidity conditions. A reduction in water vapor permeability of hydrogel biopolymer films has also been observed by others [64,65,66]. 

### 2.8. Effect of Drying Conditions and Jojoba Oil on the Microstructure of Hydrogel Whey Protein Films

Whey proteins form gels, a property currently considered their most important functional feature. This gel-forming ability is utilized in shaping the structure of food products, improving water absorption, and preventing syneresis. Furthermore, water molecules and other ingredients are retained and immobilized within the gel matrix [13]. Relative humidity positively affects the structure and surface of films based on whey protein isolate. Lipids are added to edible films and coatings to improve their properties and structure [5]. 

**Figure 7:**
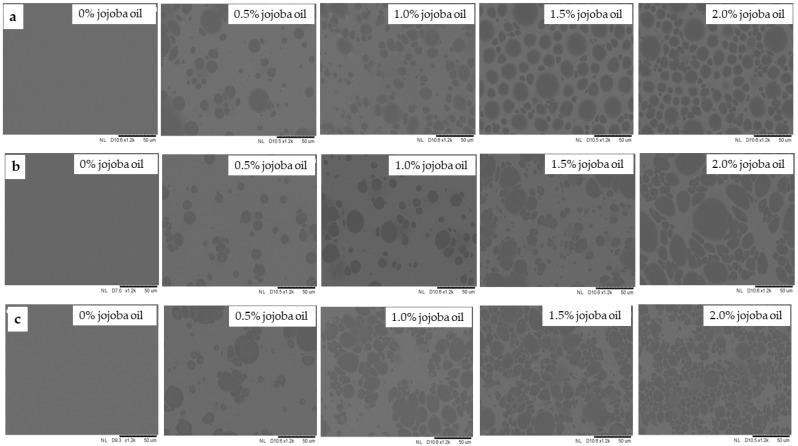
Microscopic photographs of the surface of whey protein hydrogel films incorporated with jojoba oil at the magnification of 1200×: (**a**) surface of films dried at a temperature of 20 °C and relative humidity of the environment of 30%; (**b**) surface of films dried at a temperature of 70 °C and a relative humidity of 30%; (**c**) surface of films dried at a temperature of 50 °C and a relative humidity of 70%.

**Figure 8:**
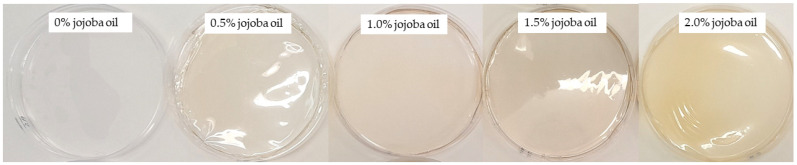
Photographs of the whey protein hydrogel films incorporated with jojoba oil dried at a temperature of 50 °C and relative humidity of the environment of 30%.

After the drying process, the structure of the prepared hydrogel whey protein films was analyzed using scanning electron microscopy. The photographs for three selected drying parameters are presented in Figure 7. Visually, films with a smooth and slightly matte surface were obtained. On the surface of the tested films, small amounts of oil were observed in the form of drops of various sizes, shapes, and irregular distribution. With increasing content of jojoba oil (0.5–2.0%) in the film-forming solutions, there was an observed increase in the amount of oil precipitated on the surface of the films. Moreover, the surfaces of whey films with added oil emulsion differed significantly from the control coating. Similar relationships were previously observed for whey films with rapeseed oil addition [50] and for casein films modified with a fatty mixture of beeswax and oleic acid [69]. Clusters of oil droplets of various sizes were observed on the surface in contact with air, indicating aggregation. The uneven distribution of oil droplets related to their mobility and tendency to agglomerate was probably influenced by drying time, indicating that longer durations could intensify this agglomeration. Upon analyzing the surface of films immediately after drying and examining microscopic photos, a detrimental effect of jojoba oil addition on the appearance of the films was observed. There were significant amounts of oil on the film surfaces and noticeable heterogeneous matting (Figure 8).

The addition of jojoba oil positively affects the phenomenon of film folding. The surfaces of samples without jojoba oil and those with 0.5% jojoba oil were primarily wrinkled. The most significant changes in the film structure’s corrugation were observed for drying parameters characterized by several hours of drying time. It can be observed that the drying temperature affected the structure of whey protein films. The oil droplets located on the surface of films dried at a temperature of 20 °C and a relative humidity of 30% were characterized by a spherical shape, smaller size, and more even distribution on the surface. The temperature of 70 °C and the relative humidity of 30% negatively impacted the distribution and shape of the oil globules. After the drying process, they acquired an irregular, egg-shaped form with various sizes and uneven distribution.

## 3. Conclusions

The drying time was significantly shorter at higher drying temperatures and also increased with higher relative humidity of the environment. The water content was lower for films dried at higher temperatures and at extreme relative environmental humidities (20 and 70 °C). As a result of the conducted research, the drying parameter that would have the most beneficial effect on all the tested properties of whey protein films is a temperature of 50 °C and a relative humidity of 30% over 3 h. The addition of jojoba oil resulted in noticeable color changes and a slight improvement in mechanical properties. Water vapor permeability decreased with the addition of jojoba oil. Observations of the structure of whey films showed an irregular distribution of oil globules and heterogeneity in their shapes. Particularly noteworthy is the influence of added jojoba oil, characterized by high thermal stability and oxidation resistance. Due to the growing interest in healthy and dietary foods, jojoba oil, as a low-calorie fatty compound, may be a future food ingredient worth examining in more detail. Moreover, the beneficial properties of jojoba oil, such as its very low oxidation rate and high-temperature resistance, encourage its use in the production of edible films and coatings as well as further research into this compound. Applying films or coatings based on whey proteins with the addition of jojoba oil can be used on products that naturally contain fats, such as meat and meat products, cheese, or nuts.

## 4. Materials and Methods

### 4.1. Materials

The whey protein isolate (WPI) of the purity min. 90% protein (BiPRO) was purchased from Davisco Foods International Inc. (La Sueur, MN, USA). Jojoba oil was supplied from Sigma ALDRICH (Lima, Peru). Anhydrous glycerol was purchased from Avantor Performance Materials Poland S.A. (Gliwice, Poland).

### 4.2. Film-Forming Hydrogel Solution Preparation

Hydrogel film-forming solutions with a concentration of 8% were obtained by mixing whey protein isolate with water using a Zelmer type 371.5 mixer. The mixtures were heated at 80 °C for 30 min using an RCT basic IKAMAG magnetic stirrer (IKA-Werke GmbH & Co., Staufen, Germany) rotating at 250 rpm. Glycerol was added in an amount of 50% relative to the added protein. The solution without adding other substances was defined as the control solution. Jojoba oil was added to the remaining parts of the solution at concentrations of 0%, 0.5%, 1.0%, 1.5%, and 2.0%. The solutions with the addition of oil were homogenized using IKA Yellowline DI25 basic (IKA-Werke GmbH & Co., Staufen, Germany) for 5 min at a speed of 24,500 rpm.

### 4.3. Particle Size and Distribution

The granulometric composition of the film-forming hydrogel solutions was analyzed using laser diffraction with a Cilas 1190 particle analyzer (Cilas, Orleans, France) at room temperature. The particle size distribution of oil globules was measured in the range from 0.04 to 2500 µm in six repetitions.

### 4.4. Film Preparation

The hydrogel solutions were poured into Petri dishes in a constant amount to obtain a similar final film thickness of 130 ± 10 µm. The materials were dried at temperatures of 20, 30, 40, 50, 60, and 70 °C with 50% relative humidity and at 50 °C with 30, 40, 50, 60, and 70% relative humidity in a climatic chamber (Binder KBF 720, Tuttlingen, Germany). After drying, the films were removed from the dishes and conditioned at 25 ± 1 °C and 50 ± 5% relative humidity for 48 h before measurements began. Drying for all parameters was performed in at least two repetitions.

### 4.5. Film Thickness

Thickness measurements of the films were conducted using an Ultrameter A400 magnetic meter (Metrison Sp. z o.o., Mościska, Poland) with an accuracy of 1 µm.

### 4.6. Water Content

Water content was determined by the drying method, in which 1 g of whey films was weighed on an analytical balance with an accuracy of 0.0001 g into previously dried weighing vessels stored in a desiccator. The prepared samples were dried at 105 °C for 24 h. Water content was determined based on the mass changes after the drying process. The study was performed in triplicate.

### 4.7. Color Measurement

The color measurement was performed using a calorimeter (Minolta, CR-300, Osaka, Japan) in the CIE L*a*b* color system, where L* represents lightness, and a* and b* are the trichromatic coordinates. The reference material was a white standard with constant values of L* (95.85 ± 0.23), a* (0.51 ± 0.06), and b* (2.59 ± 0.23). Films were placed on the standard, and measurements were made in ten repetitions. To interpret the results, the total color difference was used as a color discriminant, described by the following formula:

$$\Delta E=\sqrt{(L*-L{)}^{2}+(a*-a{)}^{2}+(b*-b{)}^{2}}$$

where ∆E is the total color difference; L*, a*, b* are the measurements for the standard; and L, a, b are the actual measurements for the films.

### 4.8. Mechanical Properties

The mechanical properties of the produced gel films were analyzed using a TA-XT2i texturometer (Stable Micro Systems, Surrey, UK) with the Texture Expert computer program. Samples measuring 25 × 100 mm were prepared for measurement, and their thickness was measured with a thickness gauge. The samples were placed between the two jaws of the device, set 25 mm apart. During the measurement, the jaws moved apart at a speed of 1 mm/s until the film broke. The system recorded the load and elongation of the film throughout this process. Based on the obtained curves, the maximum force required to tear and elongate the film was determined. Measurements of the mechanical strength of the tested gel films were performed with a minimum of eight repetitions. The tensile strength (TS) is expressed as the maximum tensile force per unit of the initial cross-sectional area of the sample’s measuring section and was calculated according to the formula:

$$TS=\frac{F_{max}}{A}$$

where TS is in MPa, F_max_ is the maximum tensile force in newtons (N), and A is the initial cross-section of the measuring section of the sample in square millimeters (mm²).

Elongation (E) of the sample was calculated with the following formula:

$$E=\frac{\Delta L}{L}\cdot 100\%$$

where E is in percent (%), L is the initial distance between the jaws in millimeters (mm), and ΔL is the increase in distance between the jaws in millimeters (mm).

### 4.9. Water Vapor Permeability

The water vapor permeability of the films was determined using the gravimetric method developed by Debeaufort et al. [70]. Circular samples were positioned between two seals and enclosed in special glass vessels featuring a hole and a twist-off cap. Distilled water was added to the vessels to maintain an environmental humidity of 100% and the corresponding partial pressure. The vessels were then placed in a climatic chamber set at a relative humidity of 30% and a temperature of 25 °C. Sample weights were measured on an analytical balance with an accuracy of ±0.0001 g, twice daily for seven days. Linear regression was used to calculate the changes in sample weight over time, omitting the initial measurements to stabilize the process conditions. The assay was performed in triplicate. Water vapor permeability was calculated using the following formula:

$$P=\frac{\Delta m\cdot e}{A\cdot \Delta t\cdot \Delta p}$$

where Δm/Δt is the weight of moisture loss per unit of time (g/s), A is the film area exposed to the moisture transfer (8.04∙10^−4^ m^2^), e is the film thickness (m), and Δp is the water vapor pressure differential between the two sides of the film (Pa). Three replicates for each film type and RH gradient were carried out.

### 4.10. Microstructure

The structural analysis was conducted using microscopic photographs of the air side of gel films (from the solvent evaporation side), captured with a scanning electron microscope (FEI Company, Quanta 200 MK2, Hillsboro, OR, USA). The test was performed using the low-vacuum method at 0.35–1 torr. Samples measuring 5 × 5 mm were affixed to the measuring table with double-sided tape of 9 mm diameter PELCO carbon paste (Pik Instruments Sp. z o.o., Piaseczno, Poland) before analyzing the selected fragment. Photographs were taken at a magnification of ×1200.

### 4.11. Statistical Analysis

The analysis of variance (ANOVA) was performed at a significance level *p* < 0.05 and with Tukey’s post hoc test to detect significant differences in film properties, using Statistica 10.0 (StatSoft Inc., Tulsa, OK, USA).

## Figures and Tables

**Table 1:** The drying time of whey films depends on the temperature and relative humidity of the environment.

Temperature (°C)	Relative Humidity (%)	Drying Time (h)
20	30	17
30	30	10
40	30	4
50	30	3
50	40	5
50	50	5
50	60	15
50	70	18
60	30	3
70	30	2

## Data Availability

The data presented in this study are available on request from the corresponding authors.
